# Roles for circulating polyunsaturated fatty acids in ischemic stroke and modifiable factors: a Mendelian randomization study

**DOI:** 10.1186/s12937-020-00582-4

**Published:** 2020-07-11

**Authors:** Tonghui Yuan, Shucheng Si, Yunxia Li, Wenchao Li, Xiaolu Chen, Congcong Liu, Jiqing Li, Bojie Wang, Lei Hou, Yanxun Liu, Fuzhong Xue

**Affiliations:** 1 Department of Biostatistics, School of Public Health, Cheeloo College of Medicine, Shandong University, Jinan, 250012 Shandong province China; 2 Institute for Medical Dataology, Cheeloo College of Medicine, Shandong University, Jinan, 250012 Shandong province China

**Keywords:** Blood pressure, Ischemic stroke, Lipids, Mendelian randomization, Omega-3 fatty acids, Omega-6 fatty acids

## Abstract

**Background:**

Available data about the effects of circulating polyunsaturated fatty acids (PUFAs) on ischemic stroke (IS) and its main risk factors remains limited and conflicting. Therefore, we conducted Mendelian randomization (MR) to assess whether genetically predicted PUFA affected IS, lipids and blood pressure (BP).

**Methods:**

Genetic instruments associated with IS were derived from ISGC Consortium (*n* = 29,633), with lipids were derived from GLGC(*n* = 188,577), with BP were derived from Neale Lab(*n* = 337,000). The inverse-variance weighted method was the main analysis to estimate the effect of exposure on outcome. Sensitivity analyses included principal components analysis, MR-Egger, weighted median, and weighted mode.

**Results:**

Per SD increases in serum α-linolenic acid (ALA) were associated with lower IS risk, with odd ratio (OR) of 0.867(0.782,0.961), arachidonic acid (AA) were associated with higher IS risk (OR: 1.053(1.014,1.094)). Likewise, Per SD increases in ALA were associated with the lower-level low-density lipoprotein cholesterol(LDL-C), high-density lipoprotein cholesterol (HDL-C), total cholesterol (TC) (*β*:-0.122(− 0.144, − 0.101), − 0.159(− 0.182, − 0.135), − 0.148(− 0.171, − 0.126), respectively), AA were associated with the higher-level of LDL-C, HDL-C and TC (*β*:0.045(0.034,0.056), 0.059(0.050,0.067), 0.055(0.046,0.063), respectively). Linoleic acid (LA), eicosapentaenoic acid (EPA), docosahexaenoic acid (DHA) and docosapentaenoic acid (DPA) had little or no association with IS, lipids or BP at Bonferroni-corrected significance. Different analytic methods supported these findings. The intercept test of MR-Egger implied no pleiotropy.

**Conclusions:**

High-level plasma ALA was protective for IS but AA was the opposite. LA, EPA, DHA, and DPA had no effects on IS.

## Background

Cardiovascular disease (CVD) was the most important contributor to the mortality and morbidity of noncommunicable diseases globally and its number of deaths made up almost one-third of total deaths worldwide [1]. People with CVD, including stroke, usually had comorbid conditions, such as diabetes, chronic obstructive pulmonary disease, and cancer, that further lowered their life quality [2]. Stroke was the second leading causes of CVD mortality, only after ischemic heart disease [1, 3]. Almost 80% of stroke was ischemic stroke (IS), which was more common compared with hemorrhagic stroke [1, 4], making it the priority of prevention of stroke. A large proportion of IS may be due to atherosclerosis [5, 6], while inflammation played an important role in it [7–9]. Polyunsaturated fatty acids (PUFAs) also influenced the process of inflammation and might or might not benefit IS [10–13]. However, the effects of PUFAs (linoleic acid (LA, 18:2ω6), arachidonic acid (AA, 20:4ω6), α-linolenic acid (ALA, 18:3ω3), eicosapentaenoic acid (EPA, 20:5ω3), docosahexaenoic acid (DHA, 22:6ω3), docosapentaenoic acid (DPA, 22:5ω3)) on IS risk continued to be inconsistent and contradictory. For example, a systematic assessment involving 17 randomized controlled trials (RCTs) found that stroke might not be affected by omega-6 fatty acids intake [11]. In contrast, another meta-analysis including 30 observational studies indicated that increased ingestion of LA was associated with a lower risk of IS risk and AA had no association with the risk of IS [14]. Similarly, although some recommendations, such as NICE Guideline, American Heart Association and so forth, suggested intake of omega-3 fats to benefit CVD [15–20], their effects that whether omega-3 fats could benefit CVD were still debated widely and varied substantially [10, 21, 22]. Several published cohort studies with different age group raised that consuming marine products (rich in EPA, DHA, DPA) was inversely associated with the risk of IS [17–19, 23]. Instead, a recent pooled-analysis of 79 RCTs substantiated that long-chain omega-3(EPA, DHA) supplements had no effect on IS, ALA had an unclear effect on stroke [10]. These conventional studies were limited by different duration, events, memory bias or confounders, so the evidence was different from each. On the other side, the research was restricted to dietary PUFA, such as supplements and marine products that were mentioned above. Thus, the estimation of PUFA may be biased by recall bias or measurement error and less precise than directly detect the PUFA concentration in the blood [24].

Yet, scattered data assessed the associations between in circulating PUFA and IS risk [25–29]. However, these observational studies were hampered by the restricted population, different methods, covariates (e.g., Physical activity, education, family income, medications), and exposure, consequently yielding controversial results. Thus, the effects of PUFA on IS risk still left vague. Furthermore, there was an amount of design applying Mendelian randomization (MR) to access the effect of one or part composition of PUFA on coronary heart disease or other cardiovascular diseases [30–32], but no analogous MR was applied to IS incidence.

To address these critical gaps in knowledge, we used MR design to test the relationships of PUFA and IS with its risk biomarkers (low-density lipoprotein cholesterol (LDL-C), high-density lipoprotein cholesterol (HDL-C), total cholesterol (TC), systolic blood pressure (SBP) and diastolic blood pressure (DBP)). MR analysis uses single nucleotide polymorphisms (SNPs) as the instrumental variables (IVs) to infer causality between exposure and outcome, which could avoid confounders and reverse causality [33]. In this study, we utilized this method to investigate the role between PUFA and IS. Additionally, lipids and blood pressure were the main modifiable factors of IS [4, 34–37]. So, we extended the objectives to these factors (LDL-C, HDL-C, TC, SBP and DBP).

## Methods

### Assumptions of Mendelian randomization

The technique of MR can be applied to evaluate the causal roles of different exposures on outcomes with IVs. It has the advantage to remove residual confounding and reverse causation. MR analysis has three assumptions: (1) IVs are related to the exposure (relevance assumption) [33]; (2) IVs share no association with any confounders of the exposure-outcome relationship (independence assumption); (3) IVs do not affect the outcome except through exposure given the confounders (exclusion restriction assumption) [38].

### Data sources

#### Genetic instruments for polyunsaturated fatty acids

In this MR analysis, six main PUFAs were selected, including two omega-6 fatty acids (LA and AA) and four omega-3 fatty acids (ALA, EPA, DHA, and DPA). Single nucleotide polymorphisms (SNPs) associated with individual fatty acids were obtained from the hitherto largest available GWAS of PUFA [39, 40]. First, we chose SNPs reached a genome-wide significance level (*p* < 5 × 10^− 8^), identifying 173,228,75,125,57 and 184SNPs relevant to LA, AA, ALA, EPA, DHA, DPA, respectively. Then, we ruled out non-bi-allelic SNPs through the SNPchip function of LDlink [41]. Next, based on the first step, we picked out SNPs in genes identified to functionally relate to fatty acid metabolism, such as FADS1, FADS2, etc. [39, 40]. Principal components analysis (PCA) was used to take account of the correlations of all significant SNPs and functionally related SNPs [42]. Besides, we also chose the top significant and independent SNPs (*p* < 5 × 10^− 8^, linkage disequilibrium (LD) r^2^ < 0.001) for the main analysis. To access the strength of instruments, we obtained variance explained by top significant and independent SNPs from the original data. We also calculated the F statistics of individual SNP [43]. If F statistics were greater than 10, then it is conceivable that these SNPs were strong instruments [44]. Detailed information for each SNP is presented in Additional file 1: Table S1–6. To guarantee chosen SNPs were relevant to IS alone, we excluded SNPs directly associated (*p* < 5 × 10^− 8^) with cardiovascular relevant phenotypes in the sensitivity analysis.

#### Genetic associations with ischemic stroke and its risk factors

Genetic associations with IS were drawn from the International Stroke Genetics Consortium (ISGC), which included 10,307 Caucasian cases and 19,326 Caucasian controls without covariates adjusted [45]. Genetic associations with lipids (LDL-C, HDL-C, TC) were derived from the Global Lipids Genetic Consortium (GLGC), which included 188,578 individuals of European lineage and adjusted for age, age^2^, and sex [46]. Genetic associations with blood pressure (SBP and DBP, automated reading) were derived from UK Biobank summary data made by Neale Lab, which includes 337,000 British individuals imputed using HRC imputation reference panel and adjusted for age, age^2^, sex, the first 20 principal components, the interactions of age^2^ with age and sex [47]. Detailed information for SNPs that were associated with Ischemic stroke and its risk factors is presented in Additional file 2: Table S1–6. Summary data sources were included in Additional file 2: Table S7.

### Statistical analysis

For the top significant and independent SNPs (*p* < 5 × 10^− 8^, LD *r*^*2*^ < 0.001), we estimated the causal effect of each PUFA with IS, LDL-C, HDL-C, TC, SBP, and DBP by inverse variance weighted (IVW) method (numbers of SNPs> 1) or Wald estimate (numbers of SNPs = 1) as our primary results. For the significant but dependent SNPs (*p* < 5 × 10^− 8^), after discarding SNPs with potential pleiotropy, we performed PCA for all remaining SNPs (*p* < 5 × 10^− 8^) and all functionally related SNPs (functionally relevant, *p* < 5 × 10^− 8^), as in previous similar research [30]. PCA weights the genetic correlation matrix, so it is robust to different SNPs selection and not subject to numerical instabilities [42]. We calculated the Correlation matrix by the TwoSampleMR R package. We also reported the original and Bonferroni-corrected results to ensure the veracity. To facilitate the following power calculation, we changed the original units of each PUFA as per standard deviation (SD). Per SD change for LA, AA, ALA, EPA, DHA, DPA, in the present study corresponded to 2.69, 1.96, 0.05, 0.30,0.89, 0.17, units in % total fatty acids [48]. All causal effects represent per SD increase in genetically predicted circulating PUFA.

### Sensitivity analyses

To access the robustness of our results, we performed several sensitivity analyses. (1) We performed a weighted median method and a weighted mode method for individual PUFA with at least 3 SNPs. The former method can return an unbiased estimate even only half the SNPs were valid instruments, the latter can give an unbiased estimate if the SNPs within the largest cluster are valid instruments [49, 50]. (2) We used the MR Egger intercept test to identify the pleiotropy, the non-zero intercept of which indicated genetic instruments could also affect outcomes addition to through PUFA [38]. (3) We used three different means to select SNPs as instruments, including choosing all significant SNPs (*p* < 5 × 10^− 8^), functionally related SNPs (functionally relevant, *p* < 5 × 10^− 8^), top significant and independent SNPs (*p* < 5 × 10^− 8^, LD r^2^ < 0.001). (4) As another technique of identifying horizontal pleiotropy, we examined the pleiotropy through the database of PhenoScannerV2 [51]. SNPs were discarded if they were associated with cardiovascular risk factors at the genome-wide significance level (p < 5 × 10^− 8^).

Bi-allelic SNPs were checked in https://ldlink.nci.nih.gov/?tab=home. Statistical power was conducted at http://cnsgenomics.com/shiny/mRnd/. Whether the instruments were associated with other phenotypes was tested by the package (phenoscanner). PCA code was presented in Additional file 3. Other statistical analyses, including the LD matrix, were carried out by R (version 3.6.1, the R Foundation for Statistical Computing, Vienna, Austria) software with the package (TwoSampleMR R package).

### Ethics approval

We used publicly available data that no ethical approval is required.

## Results

### Genetic predictors for each of PULA

We conducted three methods to select genetic predictors for LA, AA, ALA, EPA, DHA, and DPA. Taking the DPA for example. 180 SNPs were genome-wide significant and bi-allelic. We further ruled out one (rs3798719) which was not available in the LD reference panel calculated correlation matrix by TwoSampleMR R package. So, the original SNPs was 179. First, we excluded SNPs pleiotropic associated (*p* < 5 × 10^− 8^) with IS, lipids and blood pressure and obtained 179,131,133,118,177,177 SNPs as all significant instruments (*p* < 5 × 10^− 8^) for IS, LDL-C, HDL-C, TC, SBP, and DBP, respectively. We did not omit SNPs directionally related to risk factors (LDL-C, HDL-C, TC, SBP, and DBP) for IS because they might be intermediate variables on the pathway. Second, we selected SNPs in genes confirmed to functionally relate to DPA metabolism from the first step and gained 83, 49, 53,46,82,82 SNPs as functionally relevant instruments (functionally relevant, *p* < 5 × 10^− 8^) for IS, LDL-C, HDL-C, TC, SBP, and DBP, respectively. Last, we filtered top significant and independent SNPs (*p* < 5 × 10^− 8^, LD *r*^*2*^ < 0.001) from GWAS and acquired 3 SNPs for IS and its risk factors. Details of instruments in steps one and two were in Additional file 1: Table S1–6. Details of instruments in step three were in Additional file 4: Table S1–6. Details of genes confirmed to functionally related to the fatty acid metabolism were in Additional file 5: Table S1.

### Causal associations with ischemic stroke

**Fig. 1:**
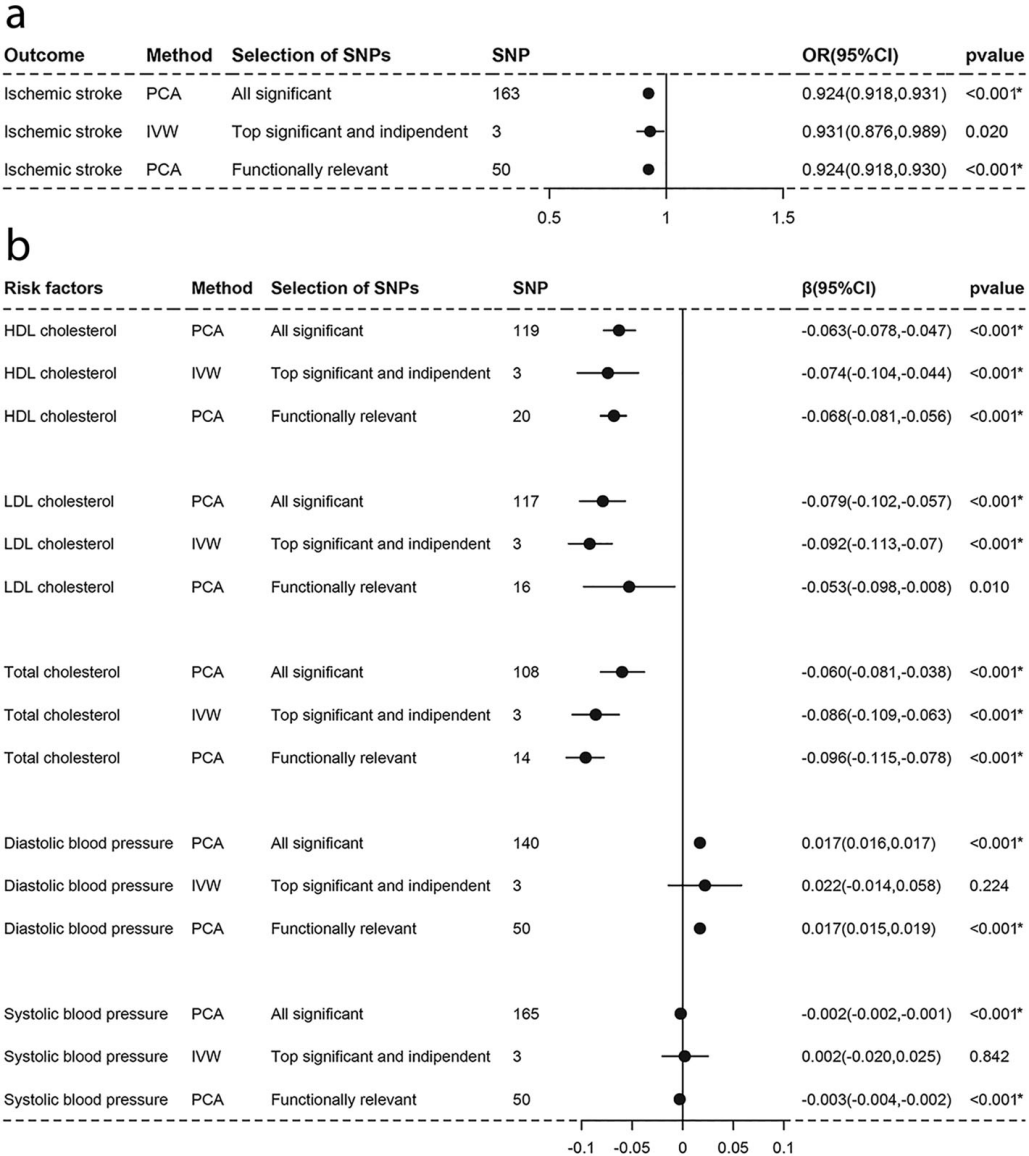
Associations of plasma LA with IS and lipids, BP estimated by IVW and PCA method. OR (95%) of ischemic stroke **a** per 1 SD increase in linoleic acid. β (95%) of risk factors **b** per 1 SD increase in linoleic acid. IVW, inverse-variance weighted. β, beta; CI, confidence interval; OR, odds ratio; PCA, principal components analysis. SNP, single nucleotide polymorphism. *Remained Bonferroni-corrected significance (*p* < 0.05/6 = 0.00833)

**Fig. 2:**
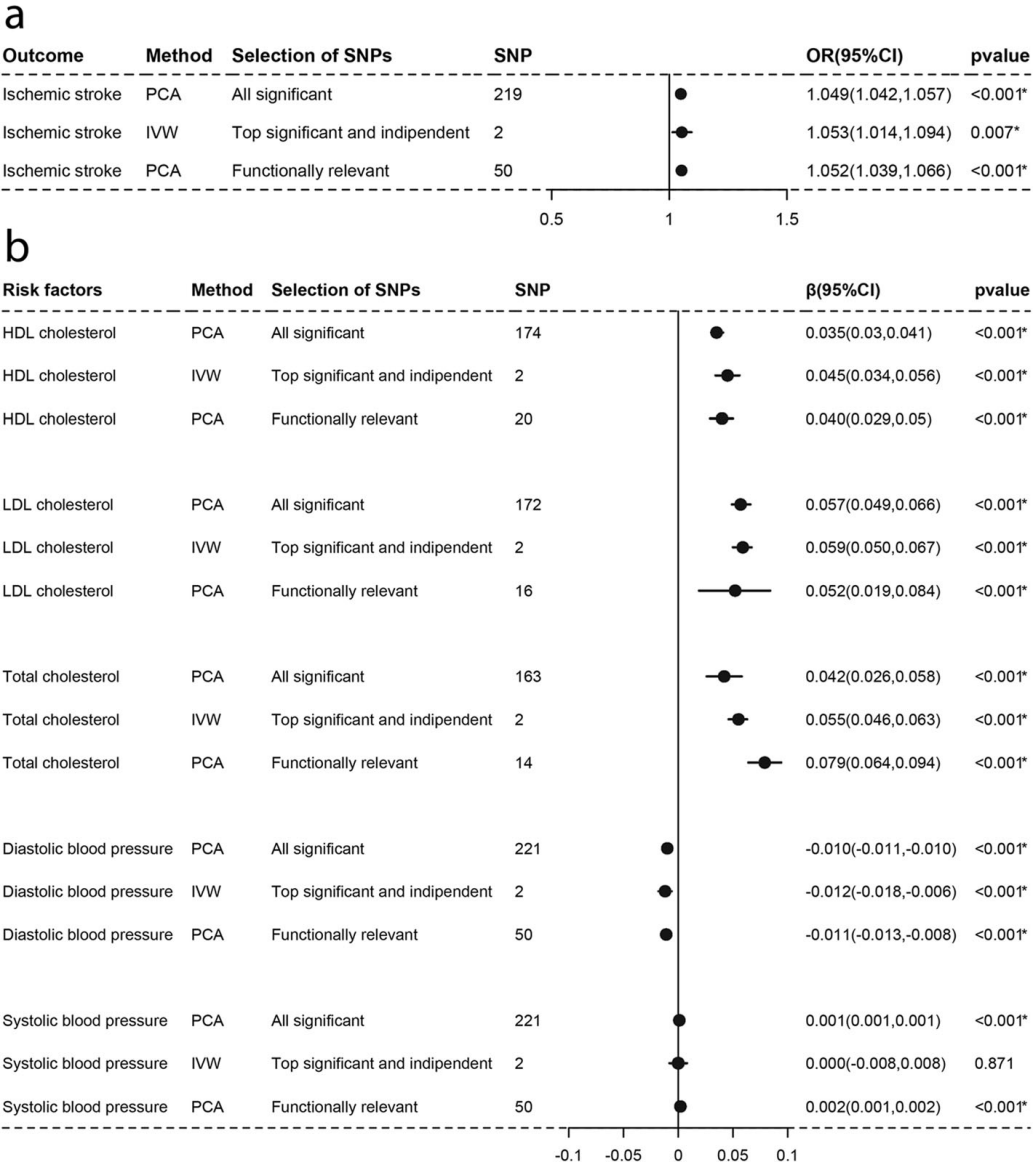
Associations of plasma AA with IS and lipids, BP estimated by IVW and PCA method. OR (95%) of ischemic stroke **a** per 1 SD increase in arachidonic acid. β (95%) of risk factors **b** per 1 SD increase in arachidonic acid. IVW, inverse-variance weighted. β, beta; CI, confidence interval; OR, odds ratio; PCA, principal components analysis. SNP, single nucleotide polymorphism. *Remained Bonferroni-corrected significance (*p* < 0.05/6 = 0.00833)

**Fig. 3:**
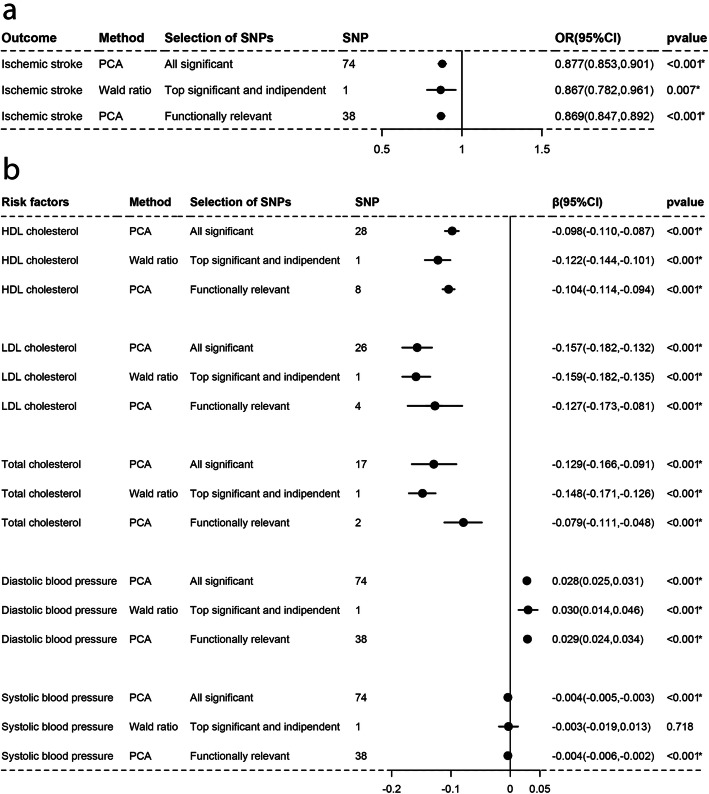
Associations of plasma ALA with IS and lipids, BP estimated by IVW and PCA method. OR (95%) of ischemic stroke **a**) per 1 SD increase in α-linolenic acid. β (95%) of risk factors **b** per 1 SD increase in α-linolenic acid. IVW, inverse-variance weighted. β, beta; CI, confidence interval; OR, odds ratio; PCA, principal components analysis. SNP, single nucleotide polymorphism. *Remained Bonferroni-corrected significance (*p* < 0.05/6 = 0.00833)

**Fig. 4:**
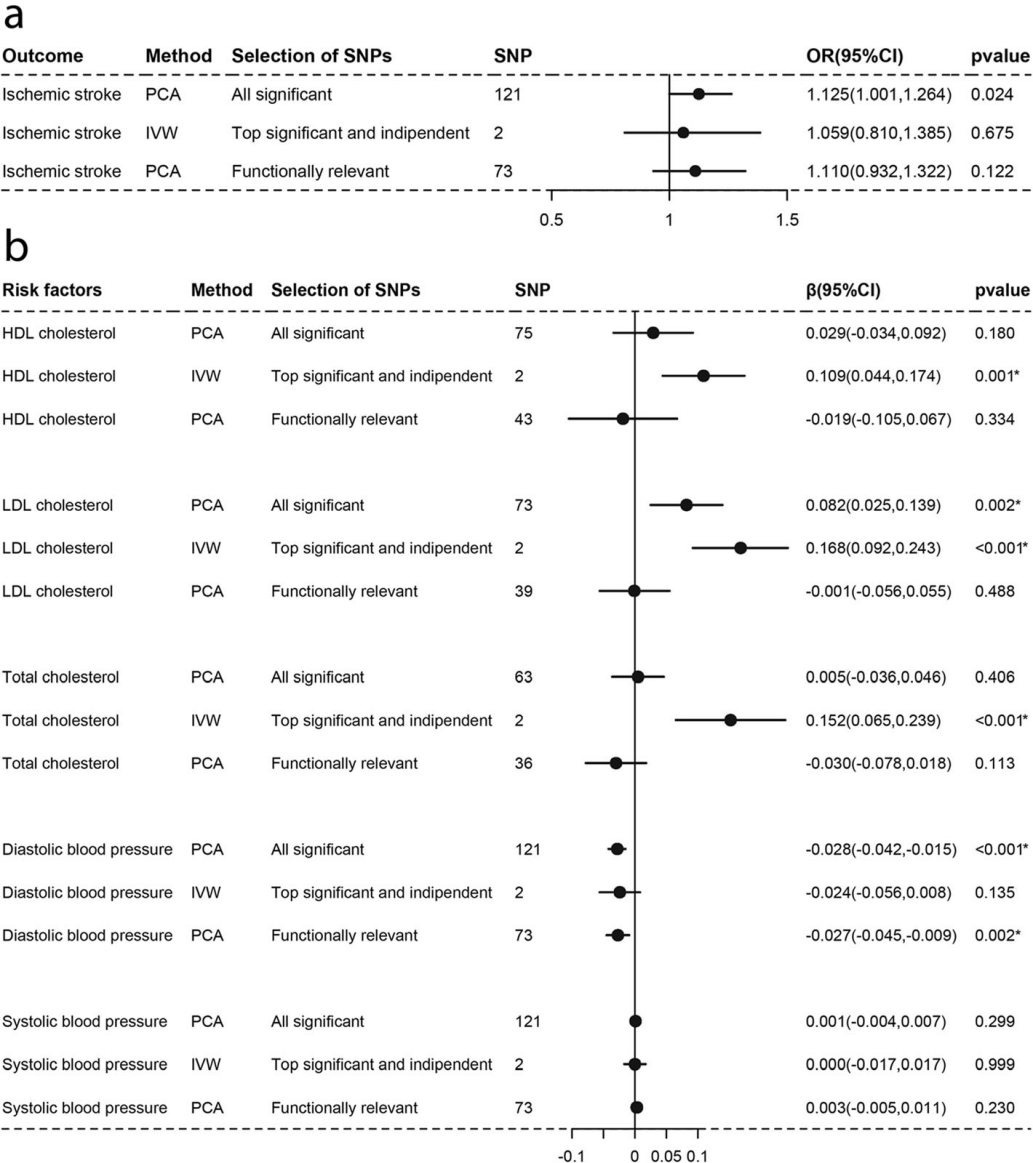
Associations of plasma EPA with IS and lipids, BP estimated by IVW and PCA method. OR (95%) of ischemic stroke **a** per 1 SD increase in eicosapentaenoic acid. β (95%) of risk factors **b** per 1 SD increase in eicosapentaenoic acid. IVW, inverse-variance weighted. β, beta; CI, confidence interval; OR, odds ratio; PCA, principal components analysis. SNP, single nucleotide polymorphism. *Remained Bonferroni-corrected significance (*p* < 0.05/6 = 0.00833)

**Fig. 5:**
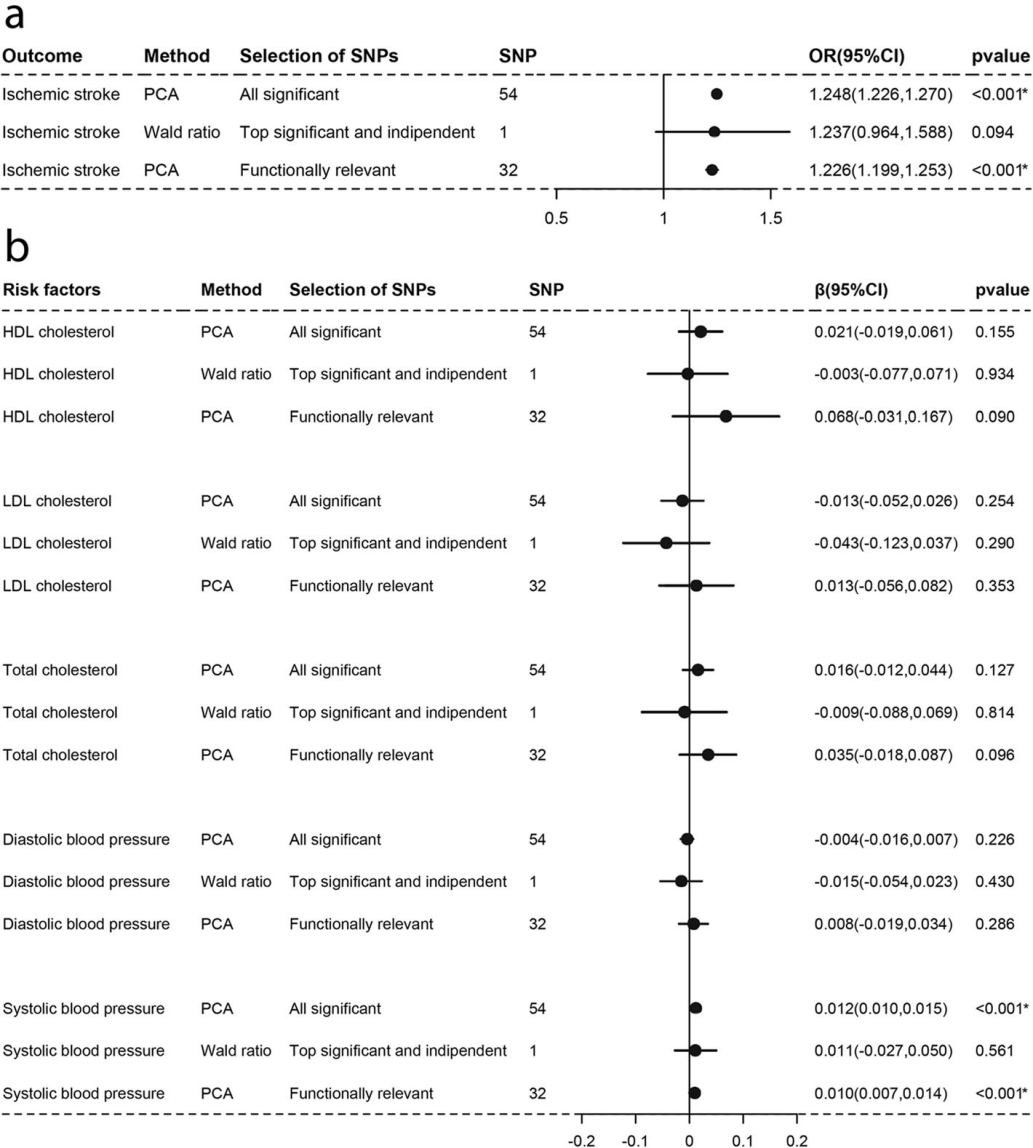
Associations of plasma DHA with IS and lipids, BP estimated by IVW and PCA method. OR (95%) of ischemic stroke **a** per 1 SD increase in docosahexaenoic acid. β (95%) of risk factors **b** per 1 SD increase in docosahexaenoic acid. IVW, inverse-variance weighted. β, beta; CI, confidence interval; OR, odds ratio; PCA, principal components analysis. SNP, single nucleotide polymorphism. *Remained Bonferroni-corrected significance (*p* < 0.05/6 = 0.00833)

**Fig. 6:**
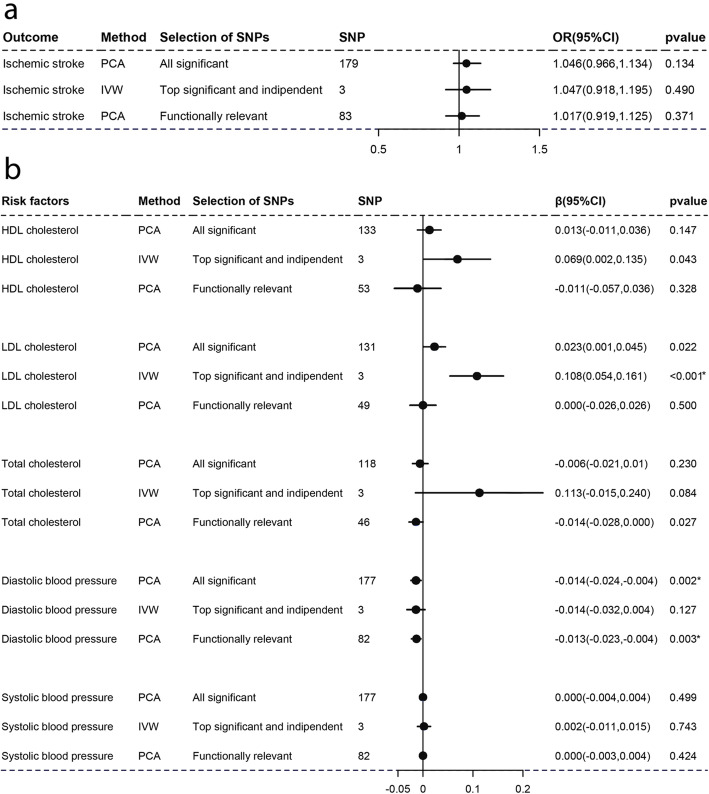
Associations of plasma DPA with IS and lipids, BP estimated by IVW and PCA method. OR (95%) of ischemic stroke **a** per 1 SD increase in docosapentaenoic acid. β (95%) of risk factors **b** per 1 SD increase in docosapentaenoic acid. IVW, inverse-variance weighted. β, beta; CI, confidence interval; OR, odds ratio; PCA, principal components analysis. SNP, single nucleotide polymorphism. *Remained Bonferroni-corrected significance (*p* < 0.05/6 = 0.00833)

As Figs. 1, 2, 3, 4, 5, and 6a-a described, results from IVW (or Wald estimate) suggested that genetic increases in ALA were negatively associated with IS incidence, while AA was positively associated with IS incidence. These results were constant with results from PCA. Genetic increases in LA, EPA, DHA, DPA could not have an association with the risk of IS as evaluations from IVW (or Wald estimate) were not concordant with PCA. Sensitivity analysis for LA and DPA were also corresponding to the above conclusions and the intercept test of MR-Egger implied our results were not affected by pleiotropy (Additional file 5: Table S2–3).

### Causal associations with lipids and blood pressure

For lipids, Figs. 1, 2, 3, 4, 5, and 6b-b indicated that genetic increments in ALA had an inverse association with HDL-C, LDL-C, and TC, AA had a positive association with them, LA only had an inverse association with HDL-C and TC, but EPA, DHA, DPA had a nonsignificant association with them, all of which was supported by IVW (or Wald estimate) and PCA. For blood pressure, IVW (or Wald estimate) and PCA remained unanimous that genetic increments in ALA were associated with higher DBP, AA was associated with lower DBP. Yet, we could not conclude that other PUFA had a relationship with DBP or SBP since at least one result of IVW (or Wald estimate) and PCA might be inconsistent (Fig. 1, 4-6b, b). Evidence from the weighted median and the weighted mode was similar to the main results. No pleiotropy was detected by the intercept test of MR-Egger for all PUFA (Additional file 5: Table S2–3).

## Discussion

In the present study, our findings suggested that genetically increased plasma ALA could lower IS risk and level of HDL-C, LDL-C, and TC. On the contrary, AA can increase IS risk and level of HDL-C, LDL-C, and TC. As for other PUFA, few or no effects were identified for their interaction with IS, lipids or blood pressure as the evidence from different methods was discordant.

The association of omega-6 fatty acids with IS and its risk factors from previous studies were nonuniform [11, 24, 29, 30, 52, 53], as some found no effects of omega-6 fats on IS, others found the protective effects [11, 14, 54]. Population-based studies found that serum LA was inversely associated with the risk of IS [14, 29, 30, 52] and HDL-C, LDL-C, TC [24, 55]. However, our MR results found that LA had no association with IS, which was supported by a systematic review of RCTs that LA did not result in increments of inflammatory metabolites [53]. Further, some research found that the effects of LA on IS might be counteracted by HDL-C and TC as LA was inversely associated with HDL-C, and TC [24, 55, 56]. In general, it might be that increase in LA reduced HDL-C and TC stimulatingly produced the counteracted effect, causing no effects of LA on IS. For another omega-6, A pooled analysis containing 30 prospective studies suggested higher AA had no association with the occurrence of IS [29]. Nevertheless, AA can be converted into pro-inflammatory markers [22], and inflammation plays a crucial role in atherosclerotic disease, including IS [5–8], which could underpin our results that higher AA was positively associated with the risk of IS, HDL-C, LDL-C, and TC. The further inference was that AA may increase IS through rising lipids level. Thus, the pro-inflammatory effects of AA might mainly influence the lipid metabolism, and further increase the risk of IS. Moreover, the pooled analysis noted that omega-6 had a neutral effect on blood pressure [24], whereas our evidence posed that AA had a lower effect on DBP, deserving further exploration. Importantly, the experiment confirmed that LA could convert into AA [13, 57]. Combining with our results, High circulating omega-6 fatty acids, especially AA, might be harmful to IS.

As for omega-3 fatty acids, their association with IS and these factors were also conflicting. Cohort investigations and reviews have preferred that intake of products rich in EPA, DHA, DPA was associated with a lower risk of IS [17–19, 23] and benefited for dyslipidemias [58, 59]. Afore meta-analysis or intervention trials highlight that the effect of ALA on IS was unknown because of low quality, on lipids (HDL-C, LDL-C, TC) [60–62] was contradictory. Because limited by different duration, small sample size, substantial quit, misclassification of PUFA, memory bias, confounding such as eating fish [61], previous views were inconsistent. However, MR had the overwhelming superiority that it could avoid confounders and inverse causality. To make clear the association between different types of omega-3 fatty acids and IS, we designed the MR research. Our results showed suggestive evidence that ALA, not EPA, DHA or DPA was of benefit in the prevention of IS, which could support some intervention trials [21, 60, 63–66]. The further inference was that ALA may lower IS through decrease lipids level. In addition, we used PUFA in vivo, which could specify which part of PUFA exerted its influence and be more precise than the dietary PUFA in previous studies.

A major strength of our design was that large GWAS databases enabled us to evaluate the association of different components of PUFA with IS and its main factors, which was more specific than the only assessment of ω-6 fatty acids or ω-3 fatty acids. Another predominant strength is that focus on circulating PUFA concentrations rather than self-reported supplementation of PUFA in the conventional epidemiological investigations could avoid memory biases and be more accurate. After all, in the conventional investigations, it is not easy to distinguish the effects which component exerts [24]. On top of the above, MR had a unique strength that using genetic predictors determined at conception as instruments could avoid reserve causality and minimize residual confounding that can bias the consequences of observational research [33].

Yet, several limitations deserve consideration. First, we had about 80% power to detect the primary results of LA, AA, and DPA on IS, but less than 50% power for evaluating ALA, EPA, and DHA because of inadequate variance (Additional file 5: Table S4). However, the evidence of ALA, EPA, and DHA was in line with the hitherto largest meta-analysis [10]. Also, our findings were applicable for the European because our samples were restricted to the European population to diminish population stratification, another ancestry is needed to increase the generalizability. Next, we attempted to conduct the multi-MR to explore the more complicated relationship between PUFA, IS and its risk factors through all significant SNPs. However, we suffered failure since SNPs were high correlated [42]. Furthermore, limited by publicly available aggregate genome-wide results, we could not explore the non-linear relationship among PUFA, risk factors and IS thought MR.

## Conclusions

In summary, our findings suggest that increases in ALA benefit for the prevention of IS but AA has the opposite effect. LA, EPA, DHA, and DPA do not affect IS. These findings are meaningful to the prevention of IS and future nutrition guideline.

## Supplementary information

**Additional file 1: Table S1.** Significant and bi-allelic SNPs (*p* < 5 × 10^− 8^) in GWAS of linoleic acid. **Table S2.** Significant and bi-allelic SNPs (*p* < 5 × 10^− 8^) in GWAS of arachidonic acid. **Table S3.** Significant and bi-allelic SNPs (*p* < 5 × 10^− 8^) in GWAS of a-linolenic acid. **Table S4.** Significant and bi-allelic SNPs (*p* < 5 × 10^− 8^) in GWAS of eicosapentaenoic acid. **Table S5.** Significant and bi-allelic SNPs (*p* < 5 × 10^− 8^) in GWAS of docosahexaenoic acid. **Table S6.** Significant and bi-allelic SNPs (*p* < 5 × 10^− 8^) in GWAS of docosapentaenoic acid.

**Additional file 2: Table S1.** Associations of SNPs (that are significant (*p* < 5 × 10^− 8^) and bi-allelic in GWAS of linoleic acid) for Ischemic stroke, LDL cholesterol, HDL cholesterol, Total cholesterol, Systolic blood pressure and Diastolic blood pressure. **Table S2.** Associations of SNPs (that are significant (*p* < 5 × 10^− 8^) and bi-allelic in GWAS of arachidonic acid) for Ischemic stroke, LDL cholesterol, HDL cholesterol, Total cholesterol, Systolic blood pressure and Diastolic blood pressure. **Table S3.** Associations of SNPs (that are significant (*p* < 5 × 10^− 8^) and bi-allelic in GWAS of a-linolenic acid) for Ischemic stroke, LDL cholesterol, HDL cholesterol, Total cholesterol, Systolic blood pressure and Diastolic blood pressure. **Table S4.** Associations of SNPs (that are significant (*p* < 5 × 10^− 8^) and bi-allelic in GWAS of eicosapentaenoic acid) for Ischemic stroke, LDL cholesterol, HDL cholesterol, Total cholesterol, Systolic blood pressure and Diastolic blood pressure. **Table S5.** Associations of SNPs (that are significant (*p* < 5 × 10^− 8^) and bi-allelic in GWAS of docosahexaenoic acid) for Ischemic stroke, LDL cholesterol, HDL cholesterol, Total cholesterol, Systolic blood pressure and Diastolic blood pressure. **Table S6.** Associations of SNPs (that are significant (*p* < 5 × 10^− 8^) and bi-allelic in GWAS of docosapentaenoic acid) for Ischemic stroke, LDL cholesterol, HDL cholesterol, Total cholesterol, Systolic blood pressure and Diastolic blood pressure. **Table S7.** Summary Statistics Data Sources.

**Additional file 3.** Software code for PCA.

**Additional file 4: Table S1.** Top significant and independent SNPs (*p* < 5 × 10^− 8^, LD *r*^*2*^ < 0.001) in GWAS of linoleic acid. Table S2. Top significant and independent SNPs (*p* < 5 × 10^− 8^, LD *r*^*2*^ < 0.001) in GWAS of arachidonic acid. Table S3. Top significant and independent SNPs (*p* < 5 × 10^− 8^, LD *r*^*2*^ < 0.001) in GWAS of a-linolenic acid. Table S4. Top significant and independent SNPs (*p* < 5 × 10^− 8^, LD *r*^*2*^ < 0.001) in GWAS of eicosapentaenoic acid. Table S5. Top significant and independent SNPs (*p* < 5 × 10^− 8^, LD *r*^*2*^ < 0.001) in GWAS of docosahexaenoic acid. Table S6. Top significant and independent SNPs (*p* < 5 × 10^− 8^, LD *r*^*2*^ < 0.001) in GWAS of docosapentaenoic acid.

**Additional file 5: Table S1.** Gene functionally relevant to the metabolism of each fatty acids. **Table S2**. Sensitivity analysis for linoleic acid (number of SNP = 3). **Table S3**. Sensitivity analysis for docosapentaenoic acid (number of SNP = 3). Table S4. Power calculation for the associations of plasma fatty acids with Ischemic stroke.

## Data Availability

ISGC https://strokegenetics.org/ GLGC http://lipidgenetics.org/#ConsortiumStudies Neale Lab http://www.nealelab.is/

## Abbreviations

ALA: α-Linolenic acid
AA: Arachidonic acid
BP: Blood pressure
DBP: Diastolic blood pressure
DHA: Docosahexaenoic acid
DPA: Docosapentaenoic acid
EPA: Eicosapentaenoic acid
GLGC: Global Lipids Genetic Consortium
HDL-C: High-density lipoprotein cholesterol
IVs: Instrumental variables
ISGC: International Stroke Genetics Consortium
IS: Ischemic stroke
LA: Linoleic acid
LDL-C: Lower-level low-density lipoprotein cholesterol
MR: Mendelian randomization
PUFA: Circulating polyunsaturated fatty acids
SNPs: Single nucleotide polymorphisms
SBP: Systolic blood pressure
TC: Total cholesterol
