# Supplementary material for: Roles for circulating polyunsaturated fatty acids in ischemic stroke and modifiable factors: a Mendelian randomization study
Source: Nutr J. 2020 Jul 11;19:70. doi: 10.1186/s12937-020-00582-4 (PMC7354684; doi:10.1186/s12937-020-00582-4)
Supplement: Supplementary file 3 — Additional file 3. Software code for PCA. [file 12937_2020_582_MOESM3_ESM.pdf]

Software code for PCA

```
#calculating LD correlation matrix by package (TwosampleMR)
ld <- ld_matrix(SNP, with_alleles = TRUE)
rho<- ld

#The associations of the candidate instruments with the risk factor are denoted betaXG with SEs sebetaXG.
#The associations of the candidate instruments with the outcome are denoted betaYG with SEs sebetaYG.

##########effect estimate using principal components###################
Phi = (betaXG/sebetaYG)%o%(betaXG/sebetaYG)*rho
summary(prcomp(Phi, scale=FALSE))
K=which(cumsum(prcomp(Phi,scale=FALSE)$sdev^2/sum((prcomp(Phi,scale=FALSE)$sdev^2)))>0.99)[1]
# K is number of principal components to include in analysis
# this code includes principal components to explain 99% of variance in the risk factor
betaXG0 = as.numeric(betaXG%*%prcomp(Phi, scale=FALSE)$rotation[,1:K])
betaYG0 = as.numeric(betaYG%*%prcomp(Phi, scale=FALSE)$rotation[,1:K])
Omega = sebetaYG%o%sebetaYG*rho
pcOmega=t(prcomp(Phi,scale=FALSE)$rotation[,1:K])%*%Omega%*%prcomp(Phi,scale=FALSE)$rotation[,1:K]
beta_IVWcorrel.pc=solve(t(betaXG0)%*%solve(pcOmega)%*%betaXG0)*t(betaXG0)%*%solve(pcOmega)%*%betaYG0

#########Q statistic (accounting for correlation) using principal components for heterogeneity:
rse = betaYG0- c(beta_IVWcorrel.pc)*betaXG0
rse.corr = sqrt(t(rse)%*%solve(pcOmega)%*%rse/(K-1))
heter.stat <- (K - 1)*(rse.corr^2)
pvalue.heter.stat <- pchisq(heter.stat, df = K-1, lower.tail = FALSE)

################# standard error for beta_IVWcorrel.pc######
se_IVWcorrel.pc=ifelse(pvalue.heter.stat>0.05,sqrt(solve(t(betaXG0)%*%solve(pcOmega)%*%betaXG0)),sqrt(solve(t(betaXG0)%*%solve(pcOmega)%*%betaXG0))*max(sqrt(t(rse)%*%solve(pcOmega)%*%rse/(length(betaXG0)-1)),1))

######### P value for beta_IVWcorrel.pc##########
P <- pnorm(beta_IVWcorrel.pc/se_IVWcorrel.pc, mean = 0, sd = 1, lower.tail = ifelse(beta_IVWcorrel.pc/se_IVWcorrel.pc<0,T,F), log.p = FALSE)
```
